# Assessment of the Effect of Structural Modification of Ibuprofen on the Penetration of Ibuprofen from Pentravan^®^ (Semisolid) Formulation Using Human Skin and a Transdermal Diffusion Test Model

**DOI:** 10.3390/ma14226808

**Published:** 2021-11-11

**Authors:** Paula Ossowicz-Rupniewska, Anna Nowak, Joanna Klebeko, Ewa Janus, Wiktoria Duchnik, Urszula Adamiak-Giera, Łukasz Kucharski, Piotr Prowans, Jan Petriczko, Norbert Czapla, Piotr Bargiel, Marta Markowska, Adam Klimowicz

**Affiliations:** 1 Department of Chemical Organic Technology and Polymeric Materials, Faculty of Chemical Technology and Engineering, West Pomeranian University of Technology in Szczecin, Piastów Avenue 42, PL-71065 Szczecin, Poland; joanna.klebeko@gmail.com (J.K.); ejanus@zut.edu.pl (E.J.); 2 Department of Cosmetic and Pharmaceutical Chemistry, Pomeranian Medical University in Szczecin, Powstańców Wielkopolskich Avenue 72, PL-70111 Szczecin, Poland; anowak@pum.edu.pl (A.N.); wiktoria.duchnik@pum.edu.pl (W.D.); lukasz.kucharski@pum.edu.pl (Ł.K.); adklim@pum.edu.pl (A.K.); 3 Department of Pharmacokinetics and Therapeutic Drug Monitoring, Pomeranian Medical University in Szczecin, PL-70111 Szczecin, Poland; uadmiak-giera@pum.edu.pl; 4 Department of Plastic, Endocrine and General Surgery, Pomeranian Medical University in Szczecin, PL-72010 Police, Poland; piotr.prowans@pum.edu.pl (P.P.); jan.petriczko@pum.edu.pl (J.P.); norbert.czapla@pum.edu.pl (N.C.); piotr.bargiel@pum.edu.pl (P.B.); markowskamh@gmail.com (M.M.)

**Keywords:** structural modification of ibuprofen, increasing drug permeability, Franz cell diffusion, transdermal delivery system, Pentravan^®^, human skin, Strat-M^®^

## Abstract

The effect of transdermal vehicle (Pentravan^®^) on skin permeability was examined for unmodified ibuprofen (IBU) and ion pairs of ibuprofen with new L-valine alkyl esters [ValOR][IBU]. The percutaneous permeation across the human skin and transdermal diffusion test model (Strat-M^®^ membranes) of ibuprofen and its structural modification were measured and compared using Franz diffusion cells. For comparison, the penetration of ibuprofen from a commercial product was also investigated. The cumulative amount of drug permeated through human skin at the end of the 24 h study was highest for ibuprofen derivatives containing propyl (C3), isopropyl (C3), ethyl (C2), and butyl (C4) esters. For Strat-M^®^, the best results were obtained with the alkyl chain length of the ester from C2 to C5. The permeation profiles and parameters were appointed, such as steady-state flux, lag time, and permeability coefficient. It has been shown that L-valine alkyl ester ibuprofenates, with the propyl, butyl, and amyl chain, exhibit a higher permeation rate than ibuprofen. The diffusion parameters of analyzed drugs through human skin and Strat-M^®^ were similar and with good correlation. The resulting Pentravan-based creams with ibuprofen in the form of an ionic pair represent a potential alternative to other forms of the drug-containing analgesics administered transdermally. Furthermore, the Strat-M^®^ membranes can be used to assess the permeation of transdermal preparations containing anti-inflammatory drugs.

## 1. Introduction

The numerous advantages of transdermal drug delivery, such as controlled or sustained drug release, constant levels of the drug in the plasma, minimizing first-pass metabolism, reduced dosing frequency, reduced drug toxicity, adverse events, and improved patient compliance, making it a convenient and frequently chosen route of drug administration [1]. Despite limiting the penetration of the desired substances into the body, the *stratum corneum* constitutes a natural protective barrier and prevents the penetration of pathogenic factors such as microorganisms or allergens and prevents excessive water loss [2,3,4]. This barrier is a thin keratin-rich layer of dead cells containing, among others, lipids [1,5,6]. Other permeation limiting factors also include physicochemical properties of the drug (solubility, molecular weight, and lipophilicity) and the properties of the drug-containing vehicle. To overcome these challenges, various attempts are made to improve the transport of drugs across the skin, such as chemical or physical methods [1]. The former includes primarily use of penetration enhancers such as among others propylene glycol [7], transcutol^®^ [8,9], terpenes [10,11,12], ethanol [13], and others.

In comparison, physical methods include, among others, microneedles [14,15], iontophoresis [16,17,18], sonophoresis [18], laser [19], and others. However, despite the various methods used to enhance penetration, it should also be taken into account. Thus, the drug permeation through the *stratum corneum* depends on the interaction between the skin, drug, and various formulation components [7].

Ibuprofen (IBU) [(*RS*)-2-(4-(2-Methylpropyl)phenyl)propanoic acid] is a commonly used nonsteroidal anti-inflammatory drug (NSAID) for the treatment of osteoarthritis and rheumatoid arthritis, as well as fever and for alleviating pain [1,2,20]. Despite the mostly chosen oral delivery route, ibuprofen is also very often applied topically to the skin. Topical use stands as an attractive alternative for conventional methods since it allows for non-invasive drug delivery and bypass of the first pass metabolism [21]. However, the therapeutic efficacy of topical application of NSAIDs is only achievable if the active substance penetrates the skin rapidly into the underlying layers [2]. The modifications of NSAID moieties for increasing their percutaneous transport are reported with growing interest [2,22,23]. Ibuprofen is characterized with low solubility in water (21 mg·dm^−3^ at 25 °C) and relative high lipophilicity (determined log P in the range of 2.41–4.00), resulting in low permeation through the skin, may also be subject to structural modification [4]. In our previous work, we presented the greater penetration compared to pure IBU of the ion pairs of ibuprofen with L-valine alkyl esters [ValOR][IBU], in which the alkyl chain R was extended from C1 to C8. For those studies alcohol such as methanol, ethanol, and isopropanol, was chosen as the vehicle. As it has worldly known the application of vehicles has a favorable impact on the penetration of the active substance [20].

Transdermal vehicles for improved transport of drugs have developed over recent years. The transdermal semi-solid vehicle should enable that incorporated drugs can reach the systemic circulation. Therefore, they often contain liposomes that assist in the transdermal delivery of the drug [24]. One such vehicle is Pentravan^®^, manufactured by Fragon (USA), an oil-in-water (o/w) emulsion base. The main component of the Pentravan^®^ is water, which accounts for 62%. Despite the relatively high water content, it appears thick, yellowish cream, with a pH of 4.0–5.5. Pentravan^®^ also includes a complex called LIPOIL, butylhydroxytoluene, simethicone, urea, potassium sorbate, polyoxyethylene stearate, cetyl alcohol, stearic alcohol, stearic acid, glycerol monostearate, benzoic acid, carbomer, and hydrochloric acid. These include substances that facilitate the transport of the drug through the skin, improve its hydration, preserve, stabilize, and improve the rheology of ointments and creams. The diversity of composition allows a cream base to prepare compounded drugs instead of local action [25]. Despite its relatively high popularity this vehicle, there are few reports of its use and the possibility of using it as a carrier of anti-inflammatory substances. The Pentravan^®^ was used as a carrier for active substances [7] in hormone or analgesic therapy [26]. There are many topical preparations on the pharmaceutical market, most of which are gel or emulsion. As shown in the literature, the penetration of ibuprofen from these preparations is very diverse [27], probably due to the physicochemical properties of ibuprofen itself and the nature of the vehicle itself. Therefore, more and more attempts are made to modify popular anti-inflammatory drugs, which, combined with a well-chosen vehicle, could give a quick and effective therapeutic effect. The advantage of increasing the lipophilicity of anti-inflammatory drugs is their faster penetration into the deeper layers of the skin and the underlying tissues, which is very important in the case of inflammation and pain. In addition, despite the wide availability of preparations on the pharmaceutical market, it is not always possible to choose the correct dose of the drug for an individual patient. Therefore, the possibility of combining a suitable vehicle with a substance with a higher permeability may be the basis for the development of transdermal preparations that act quickly and effectively.

In this work, we present the evaluation of the effect of structural modification of ibuprofen on its penetration from a Pentravan^®^ by using human skin and a transdermal diffusion test model. The purpose of introducing structural modifications of ibuprofen to the finished drug form is to increase the penetration of the drug through the skin and thus increase its bioavailability. Moreover, the influence of the vehicle on the permeability of ibuprofen and its derivatives was investigated. A commercial vehicle—Pentravan^®^—was used for the research.

## 2. Materials and Methods

### 2.1. Chemicals

Isopropanol, methanol, sodium chloride, potassium chloride, and acetic acid for all of the analyzed grades were purchased from Chempur, Piekary Śląskie, Poland, ethanol (p.a.) from Linegal Chemicals (Warszawa, Poland); acetonitrile for HPLC from J.T. Baker, Phillipsburg, NJ, USA), disodium phosphate (p.a.), potassium dihydrogen phosphate (p.a.), Strat-M^®^ membrane from Merck (Darmstadt, Germany), Pentravan^®^ from Fragon (St. Paul, MN, USA), and a commercial product from Dolorgiet (Bonn, Germany).

### 2.2. Ibuprofen and Its Derivatives

Ibuprofen (as reference material) and its derivatives—ibuprofenates of L-valine alkyl esters—were used for the research. All compounds were obtained, characterized, and described previously [2,20]. Ibuprofen and its nine L-valine alkyl esters include methyl, ethyl, propyl, isopropyl, butyl, pentyl, hexyl, heptyl, and octyl were used in the research. All derivatives were obtained by our team in accordance with the known method, starting from obtaining the hydrochlorides of the alkyl esters of amino acids, followed by neutralization and the protonation reaction with (*RS*)-2-(4-(2-Methylpropyl)phenyl)propanoic acid [2,20].

Table 1 summarizes the structural formulas, acronyms, and molar weights used ibuprofen derivatives in this research.

### 2.3. Production of Pharmaceutical Creams

**Figure 1:**
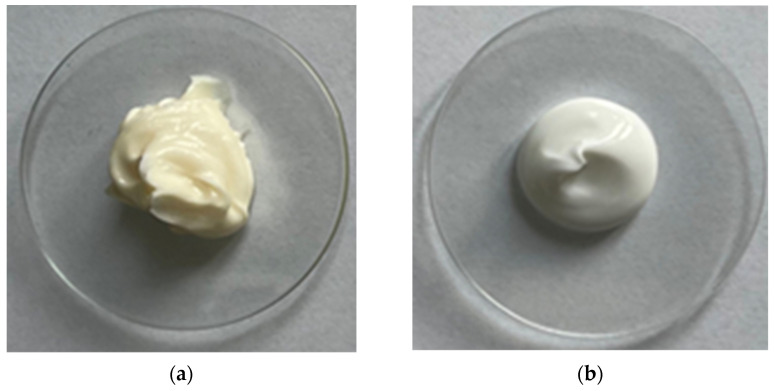
The comparison of formulation of the vehicle used (**a**) and a commercial product (**b**).

To prepare the cream, ibuprofen or its structural modifications were accurately weighed and transferred to porcelain mortar, where the levigated smooth paste was formed with a proper amount of ethanol 96% (*v*/*v*). Then, the appropriate amount of Pentravan^®^ was added to each trial and mixed for 2 min. Finally, the finished cream was transferred to the package and stored at room temperature. For comparison, a commercial preparation with 5% ibuprofen content was also used. The studies were carried out in triplicate for each compound. Compositions of the creams developed are provided in Table 2. Figure 1 presents the appearance of the formula pharmaceutical substrate, which we used in our research—Pentravan^®^ (a) and a commercially available cream containing ibuprofen (b).

### 2.4. In Vitro Penetration Studies through Human Skin and a Transdermal Diffusion Test Model

In vitro permeation studies were performed using Franz diffusion cells (SES GmbH Analyse Systeme, Bechenheim, Germany) with two types of membranes: biological membrane (human skin) and a transdermal diffusion test model (Strat-M^®^ membrane from Merck (Darmstadt, Germany)). The synthetic membranes—Strat-M^®^ with 300 µm thickness and 25 mm diameter—were used for this study. The natural tissue came from a surgical intervention after ethical approval (Ethical Committee of Pomeranian Medical University in Szczecin KB0012/02/18) and informed consent. After excision, a layer 0.5 mm thick with a dermatome and dividing into smaller pieces with dimensions of 2 cm × 2 cm, the skin was frozen at −20 °C until use, not longer than three months. This frozen storage time was safe to keep skin barrier properties [28]. Prior to use, skin samples were slowly thawed and hydrated in PBS solution at pH 7.4 for 30 min at room temperature [6,8,9]. For this research, skin samples with impedance >3 kΩ were used, which corresponds to the electrical resistance of human skin [29]. Skin impedance was measured out analogously as described previously [30].

The diffusion area of the membrane in contact with the donor and receptor phases was approximately 1 cm^2^. The volume of the donor and receptor chamber was 2 mL and 8 mL, respectively. The receptor phase was PBS solution (pH 7.4). Samples of 0.5 mL were collected at predefined times. After sampling, the same volume was replaced with a fresh buffer of the same pH. The studies were performed with 3 cells per formulation. The amount applied was about 1 g of cream (equivalent to 5000 µg of IBU). The amount of ibuprofen and its derivatives was assayed by the high-resolution chromatography method with UV detection (HPLC/UV) described below. The permeation profiles were appointed, and steady-state permeation flux (J_SS_), the diffusion coefficient (K_P_), and the time required to reach steady-state permeation (lag time—L_T_) were estimated.

### 2.5. In Vitro Membranes Accumulation

In vitro membranes accumulation was measured out analogously as described by Haq and Michniak-Kohl [13]. The membranes’ samples were removed from the receptor cells 24 h after application of the tasted creams. The membranes samples were carefully washed in 0.5% sodium lauryl sulfate solution. Following room temperature drying, each skin was weighed, cut into small pieces, placed in 2 mL of methanol, incubated for 24 h at 4 °C, and homogenized using a homogenizer (IKA^®^T18 digital ULTRA TURRAX, Staufen, Germany). The homogenate was then centrifuged for 5 min at 3500 rpm. The amount of ibuprofen and its derivatives in the supernatant were assayed by HPLC (section below) with pure methanol applied as a control.

### 2.6. HPLC Analysis

IBU and its derivatives analysis were performed by HPLC (Knauer, Berlin, Germany) using Hypersil ODS (C18) 125 mm × 4 mm column (Thermo Scientific™ Waltham, MA, USA) and a mobile phase composed of 0.02 mol·dm^−3^ potassium dihydrogen phosphate-acetonitrile-methanol (45/45/10, *v*/*v*/*v*) at 1 mL/min. The column temperature was set at 25 °C. UV detection at 264 nm was employed.

### 2.7. Statistical Analysis

The ANOVA statistical test was used to show the differences between the individual results. The significant differences were demonstrated using Tukey’s test, with **a** significance level of α < 0.05. Correlations between analyzed membranes were performed using the Pearson test. Finally, a clustering method grouped all compounds into groups with a similar penetration profile. All statistical analyses were performed using Statistica 13 PL software (StatSoft, Kraków, Poland).

## 3. Results and Discussion

The comparison of penetration of pure ibuprofen and its derivatives was presented in the previous in vitro study [2,20]. In this study, we examined the effect of the medium on the permeation of ibuprofen and its salts. Pharmaceutical creams with an active compound concentration of 5% were used as the donor phase. The acceptor phase was a buffer solution of pH 7.4. The *stratum corneum* limits the penetration of the topically applied active compounds, consisting mainly of lipids and ceramides [3], which inhibit the penetration of exogenous cosmetic and therapeutic compounds.

On the other hand, selecting the proper substrate for pairing with active substances may be crucial for increasing penetration and achieving an immediate therapeutic effect. Due to the presence of ingredients that could increase the penetration of active substances, Pentravan^®^ was used as a vehicle [5]. Another important component of the determinants the penetration of substances across the skin is lipophilicity. The lipophilicity modification of the active substances may improve penetration and achieve the preferable therapeutic concentration in the layers below [11]. Therefore, we used in the study the new derivatives characterized with hydrophilic properties (the lower lipophilicity and higher solubility in water and phosphate buffer media (pH 5.4 and 7.4) [2,20].

Our previous work showed that the obtained ibuprofen derivatives have a greater penetration skin capacity than unmodified ibuprofen from its alcohol solutions. In this research, we introduce the results of the permeability of the IBU and its structural modifications from a finished dosage form—cream, obtained based on the commercial pharmaceutical vehicle of Pentravan^®^.

**Figure 2:**
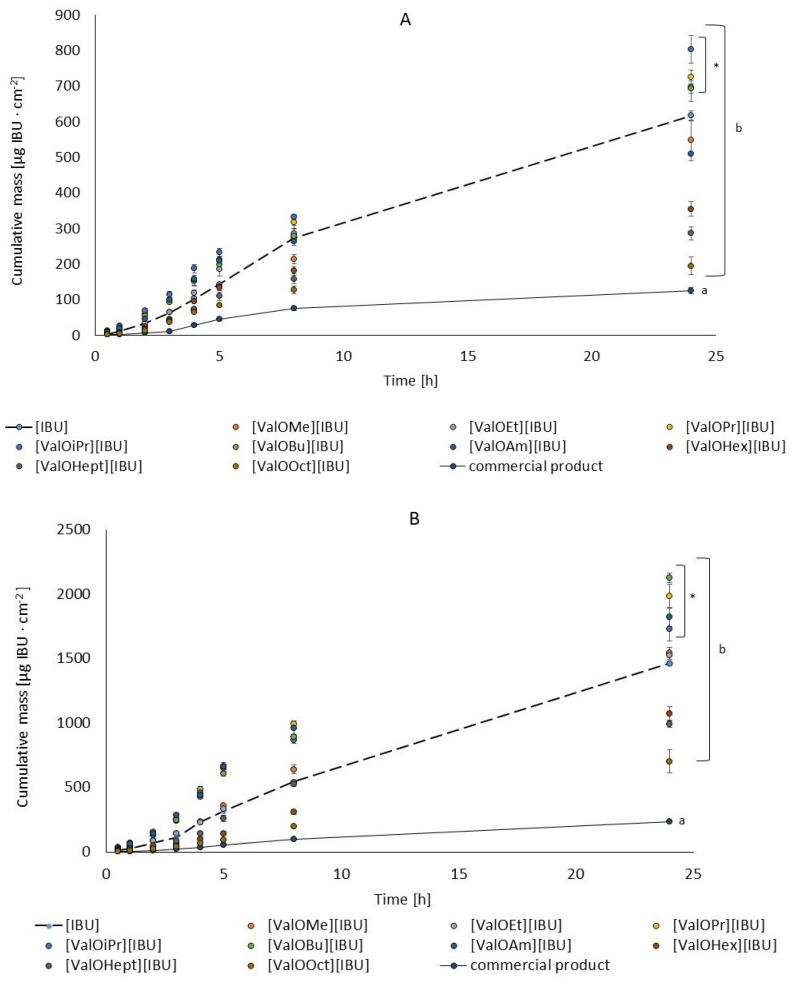
The cumulative amount of ibuprofen and its derivatives permeated human skin (**A**) and transdermal diffusion test model (**B**) as a function of time. Each point represents the mean ± SD (*n* = 3). For * *p* < 0.001 versus the control (pure ibuprofen), different letters also mean the essential between Pentravan^®^ and commercial product, where a—commercial product, b—Pentravan^®^.

The content of ibuprofen and its salts in the acceptor fluid obtained after 24 h penetration was summarized in Table 3. In contrast, the cumulative mass in acceptor fluid, including all-time points, was presented in Figure 2. Figure 2A,B compare the average cumulative mass through the entire penetration period (0–24 h). The cumulative mass of the ibuprofen in the acceptor phase after 24 h of permeation was significantly higher in application onto the human skin with the vehicle containing [ValOPr][IBU], [ValOiPr][IBU], [ValOEt][IBU], and [ValOBu][IBU] compared to the application of the acidic form. In the study with Strat-M^®^ membranes, likewise: [ValOBu][IBU], [ValOiPr][IBU], [ValOEt][IBU] and [ValOAm][IBU], and [ValOPr][IBU] compared to the application of the acidic form. When comparing ibuprofen penetration from a commercial product with a Pentravan^®^-based formulation containing an equivalent dose of active moiety, penetration from the commercial product was much lower through human skin and Strat-M^TM^ (see Table 3). The greater penetration of drugs included in Pentravan^®^ is due to its specific composition. This vehicle is a colloidal system with a hydrophilic external phase composed of isopropyl palmitate and synthetic lecithin and an aqueous phase and is a liposomal vehicle. Pentravan^®^ improves both kinetics release and bioavailability by increasing the solubility of hydrophobic drugs [31].

**Figure 3:**
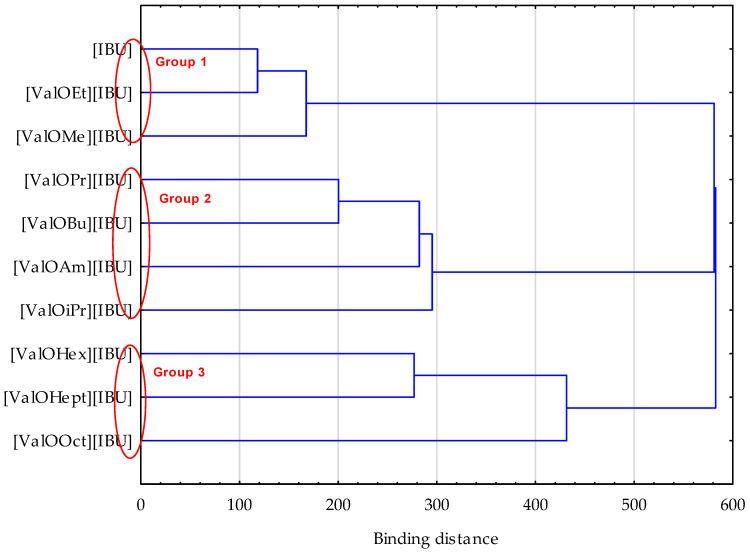
Cluster analysis plot for average cumulative mass for IBU and its derivatives permeated across human skin and Strat-M^®^.

**Figure 4:**
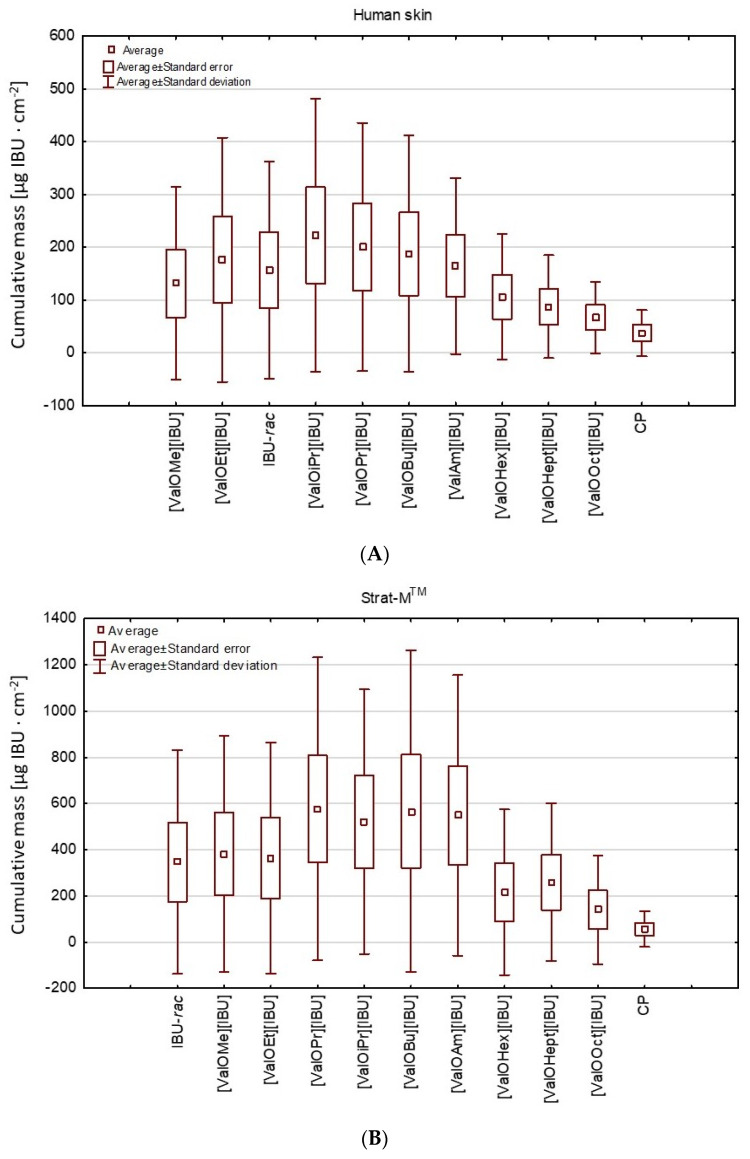
A box plot of the average cumulative mass of IBU and its derivatives after 24 h penetration through (**A**) human skin and (**B**) Strat-M^®^ membranes; CP—commercial product.

The higher penetration of ibuprofen derivatives such as [ValOiPr][IBU], [ValOPr][IBU], [ValOBu][IBU], and [ValOAm][IBU] were also confirmed by the cluster test. This test showed the similarity between these derivatives, which forms one group (red circle—group 2) (Figure 3). A lower penetration characterizes the other two groups. Similar results in comparison to free IBU were shown by [ValOEt][IBU] and [ValOMe][IBU] (red circle—group 1), while the mean cumulative mass of the active substance after 24 h permeation was the lowest for derivatives belonging to the third group (red circle) [ValOHex][IBU], [ValOHept][IBU], and [ValOct][IBU]—(Figure 3). In Figure 4, the higher penetration after a 24 h test for compounds: [ValOiPr][IBU], [ValOPr][IBU], [ValOBu][IBU] (the human skin) and additionally for [ValOAm][IBU] (the Strat-M) is also clearly visible—Figure 4. The higher penetration of some derivatives is related to their modified chemical properties. Our previous research has shown that the new ibuprofen derivatives (also used in this study) penetrated greater amounts from alcohols such as methanol, ethanol, and isopropanol [20]. These derivatives were synthesized via pairing with L-valine alkyl esters [ValOR][IBU], where R was extended from ethyl to a hexyl group [2,4]. Thus, the compounds presented in those study merge the activity of ibuprofen and amino acid. Due to the form of an alkyl ester, they increase water solubility and skin permeation of the whole compound [20]. Moreover, the salts such as [ValOiPr][IBU], [ValOPr][IBU], [ValOBu][IBU], and [ValOAm][IBU] are characterized by remarkably higher hydrophobicity compared to unmodified IBU. The saturation concentration was indicated in the range 3.055 g IBU∙dm^−3^ for [ValOEt][IBU] and 0.259 g IBU∙dm^−3^ for [ValOOct][IBU] in the buffer of pH 7.4 [20], which was also used as acceptor fluid in our study.

Wenkers et al. suggest that the skin’s permeability to anti-inflammatory drugs depends primarily on their hydrophilicity. These authors studied the penetration of several anti-inflammatory drugs, including ibuprofen from a lipophilic carrier in the form of light mineral oil. Authors suggest that the lipophilic vehicles of these drugs have a significant influence on the permeability, which can be presented as the function of their hydrophilic properties. At the same time, the maximum flux is proved to be primarily dependent on their vehicle solubilities [32].

Modifying the compound’s lipophilicity may primarily affect the penetration of active compounds into the skin. The penetration of topically applied active substances is limited by the *stratum corneum*, characterized by lipophilic properties. Therefore, the increase in the lipophilicity of the compound could make faster penetration [33,34].

The flux of ibuprofen and its derivatives across the skin was indicated from the slope of the plot of cumulative mass in the acceptor phase over time. The flux was demonstrated as the amount of active ibuprofen per skin area and time (μg∙cm^−2^∙h^−1^). As presented in Table 4, an increased rate of permeation of ibuprofen by pairing it with ValOPr, ValOBu, or ValOAm was achieved. These derivatives demonstrated the faster permeation of ibuprofen across the human skin. For the best salts, such as [ValOPr][IBU] and [ValOAm][IBU], the flux was respectively: 56.56 μg IBU∙cm^−2^∙h^−1^ and 55.10 μg IBU∙cm^−2^∙h^−1^, and it differed significantly compared to unmodified acid (36.98 μg IBU∙cm^−2^∙h^−1^). In comparison, the salts of ibuprofen with [ValOHept][IBU] and [ValOOct][IBU] showed a lower ibuprofen flux compared to the free acid. A similar trend was observed for the Strat-M^®^ membranes, where an improved significant rate of permeation for [ValOPr][IBU], [ValOiPr][IBU], or [ValOAm][IBU] was achieved. In the case of these membranes, the highest flux was obtained for [ValOPr][IBU]—205.81 μg IBU∙cm^−2^∙h^−1^ in compared control—164.45 μg IBU∙cm^−2^∙h^−1^ (Table 4). A similar result was observed in our previous studies, where the flux indicated for the ValOPr, ValOiPr, ValOBu, or ValOAm salts were higher than the control.

In other research, Sarveiya et al. reported an increased steady-state flow through a PDMS membrane by using pH 7.0 buffer as the acceptor phase for ibuprofen triethylammonium salt compared to sodium ibuprofenate [23]. On the contrary, Furukawa et al. reported a significantly higher penetration rate of ibuprofen-ProOEt through the skin of pigs compared to unmodified ibuprofen [22].

**Figure 5:**
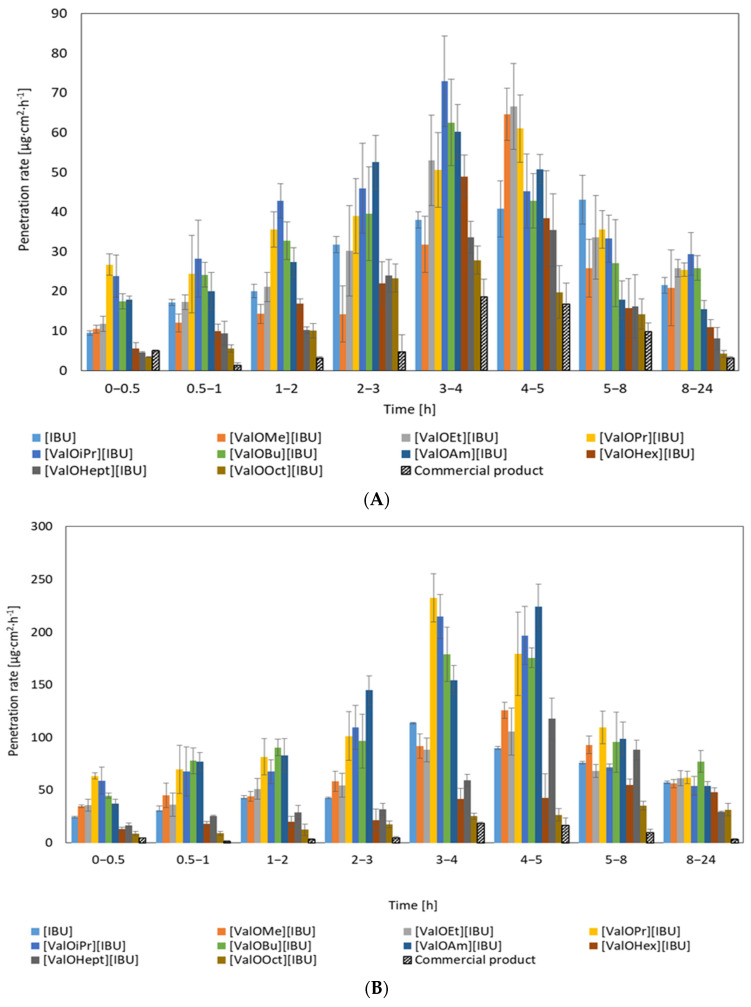
Ibuprofen’s penetration rate and derivatives permeated through human skin (**A**) and transdermal diffusion test model (**B**). Each point represents the mean ± SD (*n* = 3).

The highest penetration of studied compounds was generally observed in the time range 3 to 5 h for Strat-M^®^ membrane as well as human skin (Figure 5).

Our study used two types of membranes, namely human skin and the Strat-M^®^ synthetic membrane. Our assumption was to assess how ibuprofen’s new derivatives penetrated through the human skin. However, due to the possible high variability between the human parts, for comparison, Strat-M^®^ synthetic membranes were also used. In-vitro studies increasingly have recommended using synthetic membranes to characterize the penetration of drugs better when applied topically. Recently, the interest in synthetic artificial membranes significantly raised, among other Strat-M^®^ membranes [1,35]. The primary advantage of the Strat-M^®^ membrane shows excellent consistency and does not require any special storage, which may simplify experimental design and further data analysis [36]. In addition, human skin and the membrane Strat-M^®^ demonstrate the similar structure and chemical characteristics with a very tight top layer.

In addition, they contain a combination of lipids in a specific ratio similar to that found in the human *stratum corneum* (SC) [35]. This membrane is used in testing and optimizing pharmaceutical formulations with good reproducibility to increase confidence during early-stage drug or formulation development [35]. Furthermore, the Strat-M^®^ membrane demonstrated a better correlation to human skin with minimal lot-to-lot variability, safety, and storage limitations [1]. In this regard, Strat-M^®^ was used for our studies as an additional diffusion membrane since Uchida et al. showed a high correlation between human skin and Strat-M^®^. The authors investigated the permeation of 13 chemical compounds with a molecular weight of 152–289 and lipophilicities (log K_o/w_) of −0.9 to 3.5 [36]. In other studies, the permeation profile of rivastigmine of the synthetic membrane, Strat-M^®^, was similar to that obtained with pig ear skin [37]. In comparison, Haq et al. demonstrated the properties of Strat-M^®^, highly correlated with human skin in nicotine permeation [35]. Ibuprofen penetration testing through Strat-M^®^ membranes was previously performed by Bolla et al. [1].

**Figure 6:**
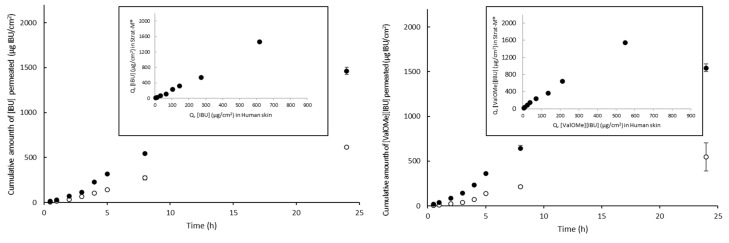

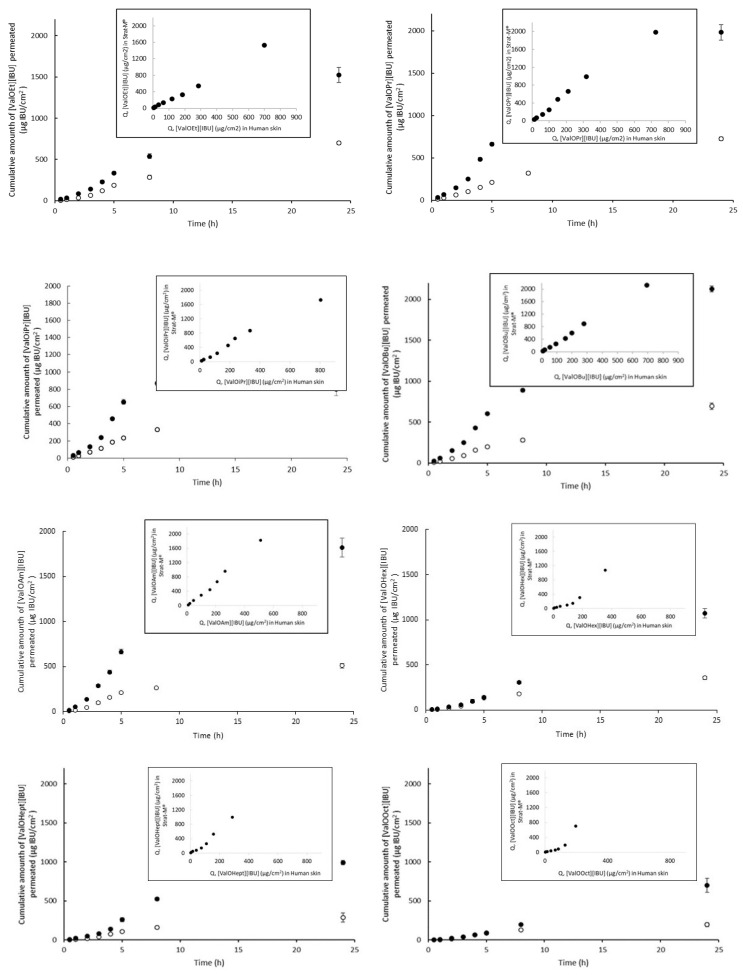
Comparison of average cumulative mass during 24 h penetration and correlation between human skin and transdermal diffusion test model for the compounds analyzed separately, Pearson’s test, *p* < 0.05.

**Figure 7:**
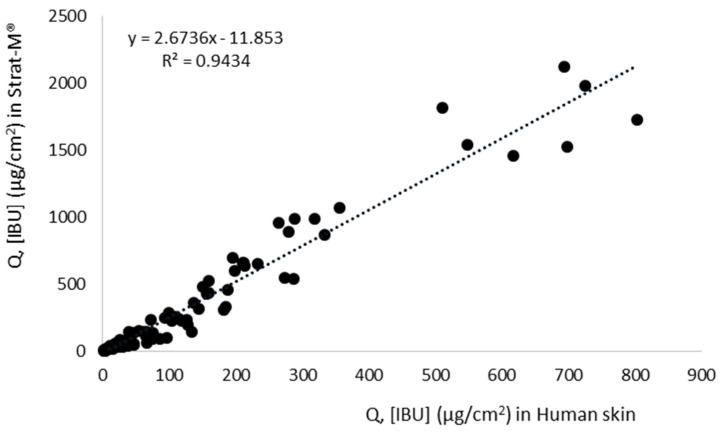
The correlation between human skin and Strat-M^®^ for all analyzed compounds, during 24 h penetration, Pearson’s test, *p* < 0.05.

In the present study, permeation experiments through Strat-M^®^ compared human skin using ibuprofen and its derivatives. The highest correlation was confirmed in the case of [ValOBu][IBU], amounting to R^2^ = 0.999, while, in other cases, the correlation ranged from R^2^ = 0.816 to R^2^ = 0.998 (Figure 6). Analyzing average penetration of all the compounds together, the correlation between human skin and Strat-M^®^ membranes was as high as R^2^ = 0.942 (Figure 7). Despite having a high correlation between Strat-M^®^ and human skin in most derivatives and free IBU, it must be noted that the amount of drug permeating through Strat-M^®^ in all cases was significantly higher than that of skin. Higher permeation through the Strat-M^®^ membrane than the natural membranes was also achieved in the case of caffeine [38], and cortisone, diclofenac sodium, mannitol, salicylic acid, and testosterone [39]. Arce et al. suggest a lack of Strat-M^®^, the highly organized intercellular structures of the SC, which in contrast are built in the skin. In this way, it does not mimic the heterogeneous complexity of the SC entirely. It fails to exhibit similar barrier properties [40]; therefore, there are frequent differences in penetration size between human skin and Strat-M^®^.

As is well-known, penetration of large molecules can be difficult. This study suggests that using an ion-pair approach enhances the permeability of the ibuprofen derivatives in the form of salts across lipophilic membranes. The ion-pair method combines a charged molecule with an oppositely charged drug molecule—this is how the charge is temporarily neutralized. Oppositely charged Coulomb forces bind the ions. Ion pairs easily penetrate the lipids of the stratum corneum and dissociate in the living layers of the epidermis [41,42,43].

**Figure 8:**
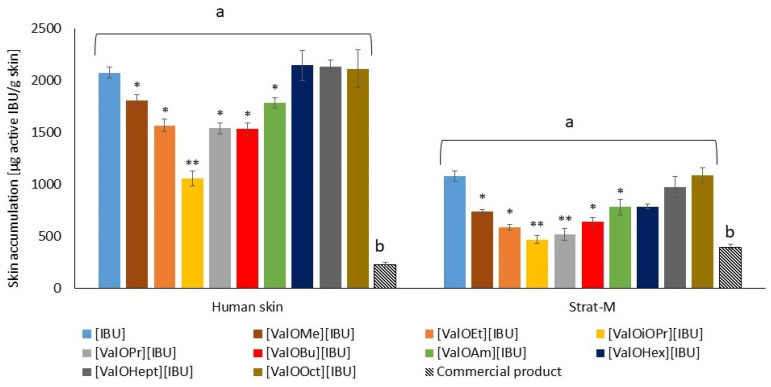
Skin accumulation results (expressed as µg of IBU per g of skin) after 24 h skin penetration of unmodified ibuprofen and its derivatives from Pentravan^®^ as well as ibuprofen from a commercial product. Points represent mean ± SD, *n* = 3. * *p* < 0.001, ** *p* < 0.0001 in compared free IBU. Different letters—important differences between Pentravan^®^ and commercial product, where a—commercial product, b—Pentravan^®^.

The drug is able to both penetrate and accumulate in the skin and Strat-M^®^. Figure 6 presents the mass of ibuprofen accumulated in human skin and Strat-M^®^ membranes in 24 h, expressed in μg IBU g^−1^ of skin. In most derivatives applied in a vehicle, a significantly lower mass of ibuprofen in the skin was observed compared with unmodified ibuprofen used. The exception was [ValOOct][IBU], [ValOHex][IBU], and [ValOHept][IBU], whose accumulation in the skin and Start-M membranes were similar compared to the control [IBU]. On the contrary, comparing the accumulated of the analyzed compounds, a significant difference was also shown between the accumulation of pure IBU and its derivatives and the commercial product used (Figure 8).

The results of our study of permeation suggest Strat-M^®^ can be an alternative to human skin for performing the skin permeation and deposition study.

## 4. Conclusions

Penetration of drug substances through the skin may be influenced, among other things, by the structure of the drug molecule and the selection of an appropriate vehicle. In our study, we estimated the penetration of free ibuprofen and its derivatives from Pentravan^®^, which is a transdermal vehicle. We also compared the penetrance of free ibuprofen with a commercial product containing this drug at the same concentration. Our studies have shown that Pentravan^®^ can be an excellent vehicle for anti-inflammatory drugs, such as ibuprofen. Compared with other commercially preparation, the Pentravan^®^ was able to deliver a higher level of drugs. Furthermore, significantly greater penetration was observed by modifying the active substance, where [ValOPr][IBU], [ValOiPr][IBU], [ValOBu][IBU], and [ValOAm][IBU] penetrated better through human skin and Strat-M^®^ membranes.

An additional advantage of using the new derivatives is the combination with L-valine, which is counted for the essential exogenous amino acids involved in many body processes, such as triggering gluconeogenesis or inhibiting the muscle-building protein degradation process. Therefore, greater penetration and the therapeutic effect of ibuprofen may have prophylactic and protective effects suitable for L-valine. Thus, applying a new transdermal vehicle with a modified drug molecule may be an exciting proposition for higher penetration and a faster therapeutic effect. Additionally, the present study of permeation suggests that Strat-M^®^ can be an alternative to human skin for performing the skin permeation of drugs.

## Figures and Tables

**Table 1:** The structural formula of ibuprofen and its derivatives.

Compound	Acronym	Structural Formula	Molar Weight [g∙mol^−1^]	Solubility in Water	log P
1	[IBU]	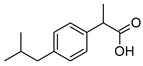	206.28	0.076 ± 0.001 [2]	2.415 ± 0.001 [2]
2	[ValOMe][IBU]	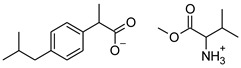	337.45	3.833 ± 0.005 [20]	0.840 ± 0.006
3	[ValOEt][IBU]	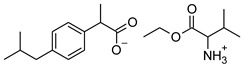	351.48	5.542 ± 0.007 [2]	0.992 ± 0.00 [2]
4	[ValOiPr][IBU]	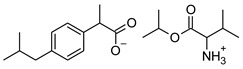	365.51	3.468 ± 0.007 [2]	1.249 ± 0.005 [2]
5	[ValOPr][IBU]	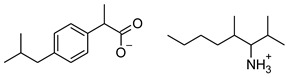	365.51	4.221 ± 0.033 [2]	1.154 ± 0.004 [2]
6	[ValOBu][IBU]	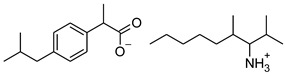	379.53	3.125 ± 0.007 [2]	1.520 ± 0.004 [2]
7	[ValOAm][IBU]	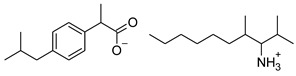	393.56	1.798 ± 0.019 [2]	1.750 ± 0.003 [2]
8	[ValOHex][IBU]	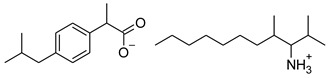	407.59	1.058 ± 0.002 [2]	1.750 ± 0.003 [2]
9	[ValOHept][IBU]	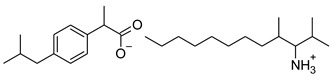	421.61	0.533 ± 0.001 [20]	2.003 ± 0.001
10	[ValOOct][IBU]	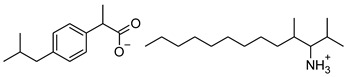	435.64	0.300 ± 0.004 [20]	2.117 ± 0.004

**Table 2:** Composition of emulsion (creams).

Compound	The Molar Mass of the Compound, g∙mol^−1^	Pharmaceutical Vehicle, g	Compound, g	Ethanol, g	TOTAL, g
[IBU]	206.284	0.8501	0.0500	0.1000	1.0001
[ValOMe][IBU]	337.458	0.8502	0.0820	0.1000	1.0322
[ValOEt][IBU]	351.485	0.8500	0.0857	0.1000	1.0357
[ValOiPr][IBU]	365.512	0.8504	0.0890	0.1000	1.0394
[ValOPr][IBU]	365.512	0.8500	0.0896	0.1000	1.0396
[ValOBu][IBU]	379.539	0.8501	0.0920	0.1000	1.0421
[ValOAm][IBU]	393.565	0.8502	0.0954	0.1000	1.0456
[ValOHex][IBU]	405.576	0.8500	0.0982	0.1000	1.0482
[ValOHept][IBU]	421.619	0.8504	0.1020	0.1000	1.0524
[ValOOct][IBU]	435.646	0.8503	0.1060	0.1000	1.0563

**Table 3:** The average cumulative mass of ibuprofen and its derivatives, expressed in mg IBU∙cm^−2^, after 24 h permeation test from the Pentravan^®^ formulation and pure ibuprofen from commercial product through human SC and transdermal diffusion test model in vitro.

Compound	Human Skin	Strat-M^®^
	Cumulative Mass (μg IBU∙cm^−2^)	Cumulative Mass (μg IBU∙cm^−2^)
[IBU]	617.263 ± 13.986 ^b^	1463.510 ± 39.201 ^b^
[ValOMe][IBU]	547.900 ± 57.800 ^b^	1540.787 ± 45.741 ^b^
[ValOEt][IBU]	698.429 ± 17.291 ^b,^*	1524.711 ± 91.932 ^b^
[ValOiPr][IBU]	725.308 ± 19.788 ^b,^*	1982.469 ± 92.275 ^b,^*
[ValOPr][IBU]	803.349 ± 39.183 ^b,^*	1732.188 ± 89.107 ^b,^*
[ValOBu][IBU]	693.593 ± 37.451 ^b,^*	2126.476 ± 37.451 ^b,^*
[ValOAm][IBU]	510.010 ± 27.905 ^b^	1822.126 ± 75.816 ^b,^*
[ValOHex][IBU]	342.336 ± 20.442 ^b^	1072.816 ± 53.024 ^b^
[ValOHept][IBU]	287.633 ± 18.271 ^b^	991.647 ± 27.137 ^b^
[ValOOct][IBU]	195.635 ± 24.639 ^b^	701.425 ± 88.702 ^b^
**Commercial product (CP)**		
	**Cumulative mass** **(μg IBU∙cm^−2^)**	**Cumulative mass** **(μg IBU∙cm^−2^)**
Ibuprofen	125.656 ± 7.827 ^a^	234.500 ± 6.703 ^a^

* Value is higher significantly from control (ibuprofen) (*p* < 0.001), different letters also mean significant differences between Pentravan^®^ and commercial product, where ^a^—commercial product, ^b^—Pentravan^®^, α = 0.050.

**Table 4:** Permeability parameters for transport of ibuprofen and its derivatives through transdermal diffusion test model and human SC in vitro.

Compound	Human Skin			Strat-M^®^			Permeation Ratio (J_Strat-M_^®^/J_Skin_)	r^2^ (Q_Strat-M_^®^ vs. Q_Skin_)
	Jss, μg∙cm^–2^∙h^–1^	K_P_·10^3^, cm∙h^–1^	L_T,_ h	Jss, μg∙cm^–2^∙h^–1^	K_P_·10^3^, cm∙h^–1^	L_T,_ h		
[IBU]	36.98 ± 1.45 ^b^	0.74 ± 0.03 ^bc^	1.17 ± 0.01 ^d^	163.45 ± 12.13 ^e^	3.26 ± 0.24 ^d^	1.55 ± 0.04 ^c^	4.42 ^e^	0.995 ^c^
[ValOMe][IBU]	36.37 ± 0.35 ^b^	0.72 ± 0.01 ^b^	1.81 ± 0.09 ^g^	82.38 ± 5.30 ^cd^	1.65 ± 0.11 ^c^	1.06 ± 0.05 ^ab^	2.27 ^b^	0.998 ^c^
[ValOEt][IBU]	43.23 ± 5.52 ^c^	0.86 ± 0.11 ^c^	1.20 ± 0.04 ^d^	77.66 ± 4.98 ^c^	1.54 ± 0.10 ^c^	0.97 ± 0.06 ^a^	1.79 ^ab^	0.995 ^c^
[ValOiPr][IBU]	43.71 ± 3.46 ^c^	0.87 ± 0.0 ^c^	0.55 ± 0.02 ^a^	177.23 ± 4.97 ^f^	3.54 ± 0.10 ^d^	1.33 ± 0.05 ^bc^	4.05 ^d^	0.993 ^c^
[ValOPr][IBU]	56.56 ± 2.74 ^d^	1.12 ± 0.01 ^d^	0.83 ± 0.12 ^b^	205.81 ± 12.78 ^g^	4.09 ± 0.25 ^e^	1.82 ± 0.02 ^d^	3.64 ^cd^	0.986 ^c^
[ValOBu][IBU]	49.73 ± 5.23 ^cd^	0.99 ± 0.01 ^d^	0.98 ± 0.01 ^c^	153.29 ± 9.75 ^e^	3.10 ± 0.20 ^d^	1.17 ± 0.02 ^ab^	3.07 ^c^	0.999 ^c^
[ValOAm][IBU]	55.10 ± 2.95 ^d^	1.09 ± 0.01 ^d^	1.17 ± 0.14 ^d^	172.48 ± 1.73 ^f^	3.42 ± 0.03 ^d^	1.28 ± 0.10 ^bc^	3.13 ^c^	0.986 ^c^
[ValOHex][IBU]	43.65 ± 3.42 ^c^	0.87 ± 0.01 ^c^	1.89 ± 0.01 ^g^	50.23 ± 1.50 ^b^	0.99 ± 0.03 ^b^	2.00 ± 0.32 ^de^	1.15 ^a^	0.912 ^b^
[ValOHept][IBU]	32.47 ± 4.89 ^b^	0.65 ± 0.01 ^b^	1.55 ± 0.09 ^f^	93.98 ± 2.83 ^d^	1.88 ± 0.06 ^c^	2.22 ± 0.17 ^e^	2.89 ^bc^	0.977 ^c^
[ValOOct][IBU]	23.80 ± 1.83 ^a^	0.47 ± 0.01 ^a^	1.35 ± 0.03 ^e^	31.96 ± 1.10 ^a^	0.64 ± 0.02 ^a^	1.95 ± 0.18 ^d^	1.34 ^a^	0.816 ^a^

J_SS_—steady-state flux; K_P_—permeability coefficient; L_T_—Lag time; r^2^—correlation coefficient. One-way analysis of variance was applied using (Tuckey’s test, α = 0.05). Different letters mean significant differences between individual substances. The letters from ‘a’ to ‘g’ were used, where a—compounds with the lowest values of penetration, and followed to g—compounds with the highest values of penetration parameters.

## Data Availability

The data presented in this study are available on request from the corresponding author.
